# Prenatal Nitrogen Oxide (NO_*x*_) and Its Potential Impact on Infant Metabolism during the First Month of Life: Evidence from Two Distinct Cohorts—The Atlanta African American Maternal-Child Cohort and the Southern California Mother’s Milk Study

**DOI:** 10.1021/acs.est.5c04955

**Published:** 2025-09-04

**Authors:** Elizabeth A. Holzhausen, Youran Tan, Nathan Young, Roshonda B. Jones, Ziyin Tang, Jeremy A. Sarnat, Fredrick Lurmann, Howard H. Chang, ViLinh Tran, Dean P. Jones, Michael I. Goran, Anne L. Dunlop, Donghai Liang, Tanya L. Alderete

**Affiliations:** † Department of Environmental Health and Engineering, 25802 Johns Hopkins Bloomberg School of Public Health, Baltimore, Maryland 21205, United States; ‡ Rollins School of Public Health, 1371 Emory University, Atlanta, Georgia 30322, United States; § Sonoma Technology Inc., Petaluma, California 94954, United States; ∥ School of Medicine, Emory University, Atlanta, Georgia 30322, United States; ⊥ 5150 Children’s Hospital Los Angeles, Los Angeles, California 90027, United States

**Keywords:** prenatal, nitrogen dioxide, fecal metabolomics, circulating metabolomics, infant

## Abstract

Exposure to traffic-related air pollution (TRAP) during pregnancy has been linked with adverse health outcomes, yet the biological mechanisms remain poorly understood. High-resolution metabolomics offers a promising approach to examine how TRAP influences infant health. However, few studies have focused on Black and Latino populations, who are disproportionately exposed to TRAP. This study aims to assess the association between prenatal exposure to TRAP and the infant metabolome in two distinct, geographically independent populations: the prospective Atlanta African American Cohort and the Southern California Mother’s Milk Study (MMS). This study provides novel evidence that prenatal nitrogen oxides (NO_*x*_), a major component of TRAP, are associated with perturbations in the infant circulating and fecal metabolome during the first month of life. We found that prenatal NO_*x*_ exposure was linked with the intensity of 8 and 16 level-1 metabolites in the ATL AA and MMS, respectively. Metabolites associated with NO_*x*_ included several involved in lipid and xenobiotic metabolism. In analyses including untargeted metabolic features, we found that prenatal NO_*x*_ was associated with perturbations in metabolic pathways including oxidative stress and inflammatory response. These findings provide novel insight into the biological mechanisms by which prenatal TRAP may influence infant health and development.

## Graphical abstract

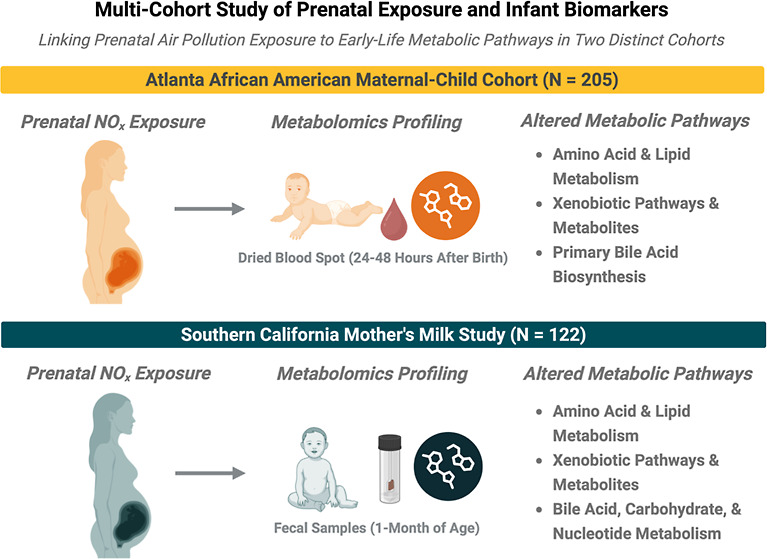

## Introduction

Nitrogen oxides are gaseous pollutants that primarily result from combustion of fossil fuels, including those used in heating, power generation, and motor vehicles.^1^ Measures of nitrogen dioxide (NO_2_) and nitrogen oxides (NO_*x*_) can serve as proxies for traffic-related air pollution (TRAP), which contains a mixture of gases and particles such as black carbon, polycyclic aromatic hydrocarbons, and particulate matter.^2^ Although motor vehicle emissions have decreased in recent years, exposure in communities can vary widely.^3,4^

TRAP exposure has been associated with adverse health outcomes such as adverse respiratory, cardiometabolic, and birth outcomes including low term birth weight and small for gestational age.^5^ Understanding how intrauterine TRAP exposure impacts childhood outcomes may be particularly important, since the prenatal period represents a critical developmental window of organogenesis, where the respiratory, immune, nervous, and cardiovascular systems are rapidly developing.^6^ Exposure to environmental hazards during this time can therefore lead to increased risk of diseases later in life. For instance, prenatal TRAP exposure has previously been associated with increased weight gain and childhood obesity,^7–9^ increased fetal glucose levels,^10^ increased risk of allergic rhinitis,^11^ and some childhood cancers.^12^

There are several potential biological mechanisms by which exposure to air pollutants may impact human health. For example, ultrafine particles can reach circulation via inhalation and diffusion from the lungs into systemic circulation.^13^ Additionally, particles may be ingested following mucociliary clearance from the airways.^14,15^ Importantly, exposure to TRAP among pregnant people can alter the placental morphology, and there is some evidence that fine particulate matter may cross the placental barrier into the fetal bloodstream.^16,17^ Thus, maternal exposure to NO_*x*_ can indirectly affect the fetus. In the neonatal period, infants are particularly vulnerable to oxidative stress, due to their immature detoxification and antioxidant systems.^18,19^

Metabolomics studies can offer novel insights into the biological impacts of TRAP exposure, including how TRAP impacts oxidative stress and inflammatory pathways, and ultimately the mechanisms underlying TRAP exposure and adverse health outcomes.^20^ Several previous metabolomics studies in adults have shown that TRAP is associated with circulating metabolites, including metabolites involved in pathways related to oxidative stress, and inflammation.^21–24^ One study explored how prenatal exposure to traffic-related PM_2.5_ (particulate matter ≤2.5 μm in diameter) impacts the metabolome of neonatal blood spots and found that prenatal TRAP was associated with metabolic pathways including lipid and amino acid metabolism, oxidative stress, and inflammation.^25^ We have also previously found that prenatal exposure to ambient air pollutants is associated with the fecal metabolome at 1 month of infant age.^26^

However, few studies have focused on communities with higher TRAP exposures. Examining communities with higher exposures is especially valuable, as the stronger exposure gradients provide a clearer lens for identifying statistically robust associations and elucidating biological pathways that, in turn, may be relevant to a broader population. Additionally, while there is some evidence that metabolic perturbations associated with air pollution are consistent across metabolomic sample types,^20^ studies which directly compare the impact of TRAP across cohorts and sample types are limited. Therefore, the aim of this study was to assess the association between prenatal TRAP exposure (estimated using nitrogen oxides, NO_*x*_) on the infant metabolome in two diverse, geographically independent populations: the Atlanta African American Maternal-Child Cohort (ATL AA) and the Southern California Mother’s Milk Study (MMS).

## Materials and Methods

### Atlanta African American Maternal-Child Cohort

#### Study Population

This project utilized data from the ATL AA Cohort, a prospective cohort that has been enrolling pregnant African American women from Grady Memorial Hospital and Emory University Hospital Midtown since 2014.^27,28^ Inclusion criteria included self-reported US-born African Americans, 18–40 years old, 8–14 weeks gestation, singleton pregnancy, ability to communicate in English, and no chronic medical conditions. Participants provided written, informed consent to participate in the study, which was approved by the Institutional Review Board at Emory University.

A total of 205 mother and newborn dyads enrolled in the ATL AA Cohort had prenatal TRAP assignments and newborn dried blood spot (DBS) metabolomics data at the time of this analysis. Sociodemographic data including maternal age and education were collected during the first clinical visit (8–14 weeks gestation), along with behavioral health information, including substance use history and current medication. Clinical data, such as maternal early pregnancy body mass index (BMI), infant sex, parity, and gestational age, were collected from patient medical records after clinical visits and postdelivery.

#### Exposure Assessment

Residential NO_*x*_ exposure for each participant was assessed using a well-established TRAP pollutant model.^29,30^ First, a calibrated Research LINE (RLINE)-source dispersion model for near-surface releases was used to estimate annual-averaged traffic-related NO_*x*_ with a high spatial resolution. Fusion modeling was then used to integrate the traffic-related NO_*x*_ data and bias-corrected simulations from the Community Multi-Scale Air Quality (CMAQ) model.^29^ This is a publicly available chemical transport model that simulates daily air pollution concentrations with a relatively low spatial resolution. The final air quality model, which incorporated comprehensive chemistry and emission sources, estimated daily ambient TRAP covering metropolitan Atlanta from 2002 to 2018 with a spatial resolution of approximately 250 × 250 m^2^.

Individual exposures were estimated by geocoding daily ambient TRAP concentrations at participants’ residential addresses collected during the first, second, and third trimester. Trimester-specific exposure was defined as the mean daily exposure from the calculated conception date, which was determined by subtracting the best obstetric estimate of the newborn’s gestational age in days from the date of the first recorded prenatal visit, to the end of the 12th week of gestation (1st trimester), from 13th to 27th weeks of gestation (2nd trimester), and from 28th to 40th weeks of gestation (3rd trimester). Cumulative exposure during pregnancy was similarly calculated until the date of birth.

### Untargeted High-Resolution Metabolomics

Within 24–48 h after birth, DBS samples were collected via heel stick using a standardized protocol, where the heel skin was first cleaned with 75% isopropanol (excess alcohol was wiped off, and the skin was allowed to air-dry). A sterile 2.5 mm lancet was then used to obtain the sample, and the sample was collected onto a standard Guthrie card by saturating each circle with approximately 75 μL of blood.^31^ Card specimens were transported to the Georgia Department of Public Health Laboratory on the day of collection and were stored (2–8 °C) without a desiccant for up to three months, at which time they were transferred to an Emory University Laboratory and stored in a −80 °C freezer inside gas-impermeable bags with desiccant until assay.^31^ For the current study, single 15 mm punches (equivalent to ∼50 μL whole blood) were collected from 279 children between 2016 and 2020.^32^ An additional set of 15 mm punches was obtained from adjacent portions of filter paper from the same Guthrie cards to use as blanks.

The North Carolina HHEAR Hub in UNC Nutrition Research Institute, North Carolina Research Campus (Kannapolis, NC) conducted untargeted high-resolution metabolomics profiling using a single run and established methods.^33^ Briefly, DBS samples underwent extraction with 1 mL ice-cold methanol, including 500 ng/mL l-tryptophan-d5. The samples were vortexed at 5000 rpm for 10 min and sonicated for an additional 20 min. After centrifugation at 16,000 rcf for 10 min at 4 °C, 600 μL of the extracted supernatant were transferred into a 2.0 mL low-bind microfuge tube. A combined 70 μL of the extracted supernatant from each of the study samples was pooled together and then redistributed into new set of tubes with 600 μL per tube to generate total study pool samples. Blanks were processed identically to study samples. The supernatant (70 μL) from each of the blanks was mixed and then distributed into multiple low-bind 2.0 mL tubes, each containing 600 μL. All supernatant aliquots (600 μL), including study samples, study pools, and blanks, were dried by vacuum concentrator overnight and reconstituted with 100 μL water–methanol (95:5, v/v) for ultrahigh performance (UHP) LC-HR-MS analysis. External quality control samples consisted of 50 μL aliquots of NIST reference plasma (SRM 1950) and were prepared using a similar procedure. After study sample randomization, quality control study pools, NIST references, and blanks were interspersed at a rate of 10% among the study samples during the analysis sequence, which was divided into three batches.

The untargeted data was acquired on a Vanquish UHPLC system coupled with a Q Exactive HF-X Hybrid Quadrupole-Orbitrap Mass Spectrometer (Thermo Fisher Scientific, San Jose, CA). A volume of 5 μL was injected for analysis. Metabolites were separated via an HSS T3 C18 column (2.1 × 100 mm, 1.7 μm, Waters Corporation) at 50 °C with mobile phases of water (A) and methanol (B), each containing 0.1% formic acid (v/v). The UHPLC linear gradient began with 2% B, and increased to 100% B in 16 min, then held for 4 min, with a flow rate at 400 μL/min. The untargeted metabolomics data was acquired in a range of 70–1050 *m*/*z* using the data dependent acquisition mode for the MS/MS spectra data.

The UHPLC-HR-MS data were processed by Progenesis QI (version 2.1, Waters Corporation) for peak identification and alignment. Background signals were excluded if the mean intensity across blanks were higher than that across the quality control study pools based on the unnormalized data.^34^ The remaining peaks were normalized by Progenesis QI using the “normalize to all” feature. Signals that significantly differed among the three running batches (ANOVA, with false discovery rate (FDR) correction *q* < 0.05) were excluded from further analysis. Finally, signals were filtered to only include those detected in 90% of serum samples and the metabolomics data was log_2_-transformed to normalize positive skewness.

### Southern California Mother’s Milk Study

#### Study Population

The MMS is a longitudinal cohort of maternal-child dyads recruited from Los Angeles maternity clinics between 2016 and 2019.^35^ Eligibility criteria included: ≥18 years of age and approximately 1 month postpartum at time of enrollment, had a healthy, singleton birth, were primiparous, and had the ability to read at a fifth grade level in either English or Spanish. Exclusion criteria included: known diagnoses impacting mental or physical health, reported use of tobacco or recreational drugs, had infants who were preterm (born prior to 37 weeks’ gestation) or low birth weight (birth weight <2500 g), or had infants who had any clinically diagnosed abnormalities. This study was approved by the Institutional Review Boards at the University of Southern California, Children’s Hospital Los Angeles, and Johns Hopkins University.

### Study Design

Overall, 219 participants were recruited at approximately 1 month postpartum and attended follow-up visits at 6-, 12-, 18-, and 24 months of infant age. At the initial visit, participants provided residential histories and detailed health and behavior information including maternal education and maternal prepregnancy BMI, both of which were assessed by questionnaire. Additional funding supported the analysis of infant fecal metabolomics samples, and 122 infant fecal samples were included for fecal metabolomics analysis at 1 month of age.

#### Exposure Assessment

As previously described,^36^ prenatal residential address histories were collected and geocoded using the Texas A&M Geocoder (http://geoservices.tamu.edu/Services/Geocode/). The total NO_*x*_ was a combination of local ambient NO_*x*_ concentration estimates from the EPA’s Air Quality system observations and the dispersion model estimates of contributions from local traffic activity.

Total NO_*x*_ was considered a proxy for exposure to the complex mixture of particles and gases emitted by motor vehicles. Information regarding the date of conception was not available. Therefore, prenatal total NO_*x*_ exposure was estimated based on average exposure for the 9 months prior to the infants’ birth. Trimester-specific estimates were exposure averages from [9, 6) months prior to birth for the first trimester [6, 3) months prior to birth for the second trimester, and [3, 0) months prior to the birth.

### Untargeted High-Resolution Metabolomics

Infant stool samples were collected at 1 month of infant age using OmniGene GUT kits. Stool samples were sent to the Emory Clinical Biomarkers Laboratory for untargeted high-resolution metabolomics profiling as previously described.^24,33^ Stool samples were added to ice-cold acetonitrile for 30 min to precipitate proteins and were then centrifuged for 10 min at 14,000 g and kept at 4 °C until analysis. All extractants were then examined using high-resolution mass spectrometry (LC-HRMS) (Dionex Ultimate 3000, Thermo Scientific Orbitrap Fusion). Two columns were used: hydrophilic interaction liquid chromatography (HILIC) (Waters XBridge BEH Amide XP HILIC column; 2.1 × 50 mm^2^, 2.6 μm particle size) with positive electrospray ionization (ESI) and reverse phase (C18) chromatography (Higgins Targa C18 2.1 × 50 mm^2^, 3 μm particle size) with negative ESI. In the HILIC chromatography column, analyte separation was done using water, acetonitrile, and 2% formic acid mobile phases following gradient elution. The initial period of 1.5 min consisted of 22.5% water, 75% acetonitrile, and 2.5% formic acid followed by a linear increase to 75% water, 22.5% acetonitrile, and 2.5% formic acid at 4 min. This was followed by a final hold for 1 min. In the C18 chromatography column, analyte separation was conducted using water, acetonitrile, and 10 mM ammonium acetate mobile phases, where the initial 1 min period used 60% water, 35% acetonitrile, and 5% ammonium acetate with a linear increase to 0% water, 95% acetonitrile, and 5% ammonium acetate at 3 min, followed by a final hold for 2 min. For both columns, the mobile phase flow rate was 0.35 mL/min for the first minute and 0.4 mL/min for the last 4 min. LC-HRMS was run in full scan mode, with 120k resolution and a range of mass-to-charge ratio (*m*/*z*) from 85 to 1275. In the HILIC chromatography column, tuning parameters for sheath gas were set to 45 (arbitrary units), auxiliary gas was set to 25 (arbitrary units), and spray voltage was set to 3.5 kV. In the C18 chromatography column, auxiliary gas was set to 30, auxiliary gas was set to 5, and spray voltage was set to −3.0 kV. Internal standards included pooled stool and standard reference materials for human stool metabolites. Internal standards were included at the beginning and end of each 20-sample batch.

Data from the HILIC and C18 columns were analyzed separately. Metabolomic signals were extracted and aligned using *apLCMS*, with a modification of *xMSanalyzer* used for quality control and reduction of batch effects.^37,38^ Metabolite intensities were measured in triplicate. Features whose intensity had a coefficient of variation (CV) greater than 30% were removed from analysis, as were metabolites detected in less than 10% of samples. Intensities of metabolic features were then averaged across the triplicates. Metabolomic features were annotated according to the Metabolomics Standards initiative criteria, and Level-1 confidence was assigned to metabolic features whose *m*/*z* and retention time matched (within 10 ppm and 50 s) authentic standards analyzed with MS/MS under identical conditions.

### Statistical Analysis

All statistical analyses described below were conducted independently in the ATL AA and the MMS. Prior to analysis, intensities of all metabolic features were log_2_-transformed to approximate normality. Descriptive analyses of key variables from both cohorts were conducted using R version 4.4.1.^39^ To assess the relationship between prenatal NO_*x*_ exposure and Level-1 metabolites, we used the *vegan* package to implement PERMANOVA analyses with Euclidian distance as our distance metric.^40^ Next, we assessed the association between prenatal NO_*x*_ exposure and the logged-intensity of Level-1 metabolites using linear models. All models were adjusted for infant age, infant sex, mother education, mother BMI, and season of study visit. Models for the Atlanta ATL AA Cohort were additionally adjusted for alcohol, tobacco, and marijuana use during pregnancy. Models fit for the MMS did not adjust for these factors because participants who reported using alcohol, tobacco, or drugs were not eligible for the study.

Models identical to the Level-1 models described above were run to estimate the association between prenatal NO_*x*_ exposure and untargeted metabolic features in both cohorts. A similar analysis was conducted but restricted to only metabolic features that were observed in both cohorts. We used *Metapone,* an innovative bioinformatic package in R to assess which metabolic pathways were associated with prenatal NO_*x*_ exposure among all metabolic features in each cohort, and among only metabolic features observed in both cohorts.^41^ Briefly, *Metapone* jointly assesses the associations among metabolites in the HILIC and C18 columns with prenatal NO_*x*_ exposure.^41^ *Metapone* uses a weighted gene set enrichment analysis (GSEA) which was modified for untargeted metabolomics data, employing established online MS databases of metabolites to putatively annotate metabolic features. Multiple testing is adjusted using a local false discovery rate (lfdr), which is a Bayesian approach that has minimal assumptions and does not rely on the assumption that all statistical tests are independent.^42^ All *Metapone* analyses used GSEA (parameter use.gsea = T) and focused on metabolites (parameter use.meta = T). Finally, we restricted both data sets to include only metabolites which were identified in both cohorts. Specifically, we used the *find.Overlapping.mzs*() function from the *xMSanalyzer* package to identify potentially overlapping *m*/*z* features between the two cohorts. Though Level-1 metabolite identification in each cohort was conducted using a 10 ppm mass tolerance against in-house authentic standard libraries, following protocols established by the Emory Clinical Biomarker Discovery Laboratory, cross-cohort feature matching used a more relaxed threshold of 30 ppm for *m*/*z* and 50 s for retention time (RT). This approach was designed to accommodate inherent variability introduced by differences in biospecimen types (DBS vs fecal), LC–MS platforms, and lab-specific analytical conditions. Importantly, the goal of matching features across cohorts was not to confirm chemical identity but to perform pathway-level analysis, which does not require precise annotation of each feature. RT alignment was particularly challenging. In the Atlanta cohort, RT was recorded in minutes rather than seconds, further complicating alignment. Previous validations of xMSanalyzer have shown that features often elute within ±60 s of predicted RT, supporting the use of a 50 s RT window in our study to account for this variability while minimizing false negatives.^33,38,43^ Putative pathway analyses were then conducted using *Metapone* in each cohort to identify metabolic pathways associated with prenatal NO_*x*_ exposure.

## Results

### Study Population Characteristics

In the ATL AA Cohort, 6,560 metabolic features were detected in one chromatography column from the newborn DBS after removal of features with CV > 30 and <10% detection (*n* = 7,228). Of the remaining features, 234 could be identified with Level-1 confidence. In the MMS, there were 11,345 metabolic features in the infant fecal metabolome extracted from the HILIC chromatography column and 8,609 from the C18 chromatography column after removal of features with CV > 30 (*n* = 1,325 and 835 in the HILIC and C18 columns, respectively) and features with <10% detection (*n* = 712 and 581 in the HILIC and C18 columns, respectively). Of the remaining features, 143 from the HILIC column and 104 from the C18 column could be identified with Level-1 confidence, where metabolic features were matched using *m*/*z* and RT to authentic standards with MS/MS under identical conditions (Figure 1).^44^

**Figure 1:**
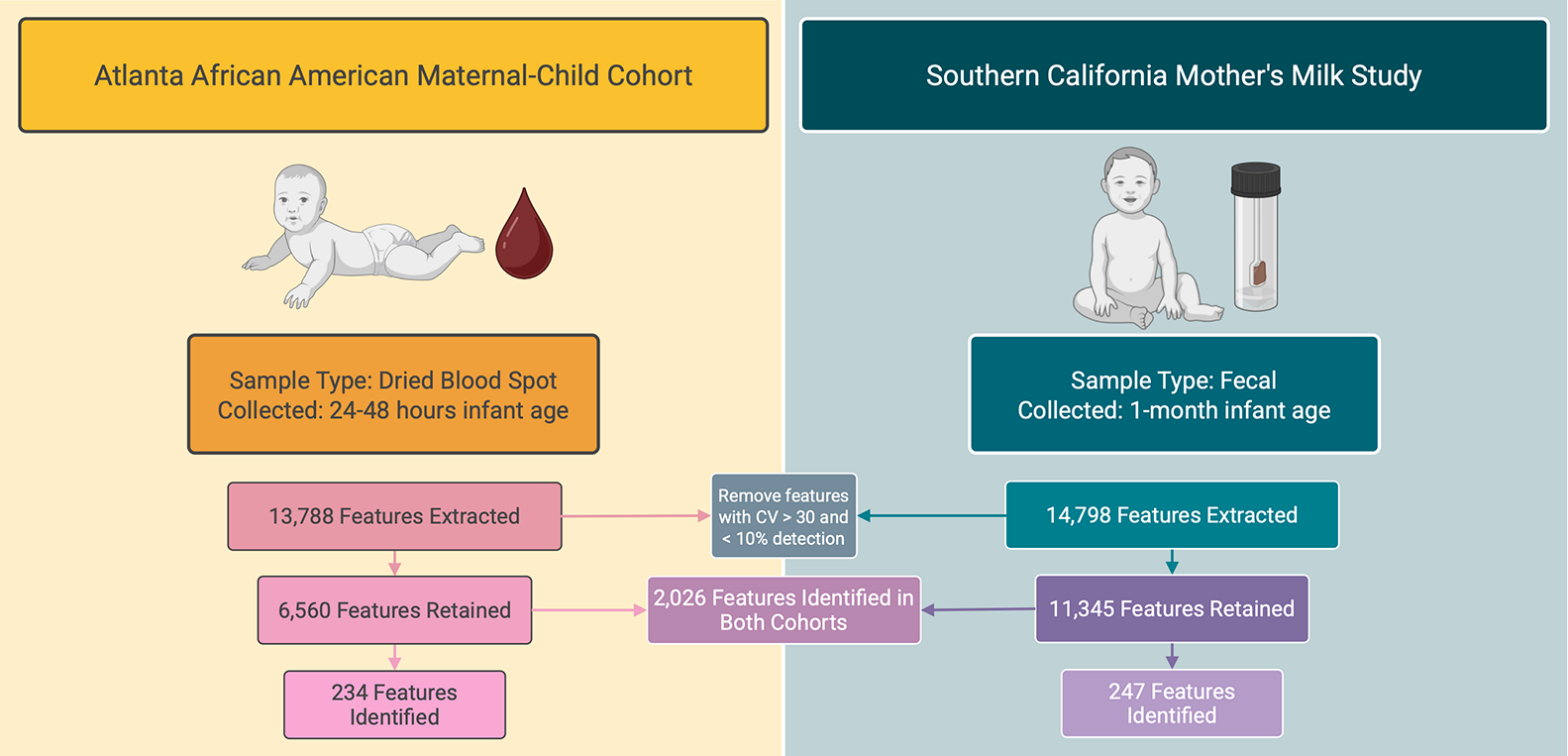
A summary of the metabolic features extracted, retained, and identified in the Atlanta African American Maternal-Child Cohort and the Southern California Mother’s Milk Study. Figure created using Biorender.com. Abbreviations: CV = coefficient of variation.

Participant characteristics from each cohort are described in Table 1. In the ATL AA Cohort, mothers were 25 ± 5 years old at the time of the infant’s birth (range: 18–40 years), 57% had parity ≥1, and 36% reported taking antibiotics. In the MMS, mothers were 29 ± 6 years old at the 1 month study visit (range: 18–45 years) and 9% reported taking antibiotics. Metabolomics in the ATL AA Cohort were measured via DBS, which were collected within 48 h of delivery. Therefore, infants in this cohort were between 0 and 2 days of age, while infants in the MMS were 33 ± 3 days old at the time of fecal metabolomics assessment. In both cohorts, infants were 46% male. In the ATL AA Cohort, 18% of infants were born by caesarean section compared to 27% of infants in the MMS. The ATL AA Cohort was originally convened to assess the associations between the prenatal microbiome environmental toxicants with preterm birth. Therefore, 23% of infants were born preterm (i.e., between 22 and 0/7 and 36–6/7 weeks gestation), 58% were early term (between 37 and 0/7 and 38–6/7 weeks gestation), and 19% were full term or late (39–0/7 weeks or greater). Potential mother-infant dyads from the MMS were excluded from participating if infants were born preterm; 3% of infants were born early term and 97% were born full or late term based on maternal self-report.

**Table 1:** Characteristics of Participants in the Atlanta African American Maternal-Child Cohort (2014–2020) and the Southern California Mother’s Milk Study (2016–2019)

characteristic	Atlanta African American Maternal-Child Cohort *N* = 205	Southern California Mother’s Milk Study *N* = 122
	mean ± SD or *N*, %	mean (SD) or *N*, %
Maternal Characteristics		
age (years)	25.1 ± 4.8	28.9 ± 6.3
body mass index (kg/m^2^)^a^	28.8 ± 7.7	28.7 ± 5.8
education	30 (15%)	39 (32%)
<high school	88 (43%)	66 (54%)
high school or some college	54 (26%)	12 (10%)
college degree	33 (16%)	5 (4%)
graduate degree		
parity (0, 1+, %1+)^b^	89, 116, 57%	
alcohol use during pregnancy (yes, no, % yes)^c^	21, 184, 10%	
tobacco use during pregnancy (yes, no, % yes)^c^	35, 170, 17%	
marijuana use during pregnancy (yes, no, % yes)^c^	84, 121, 41%	
season of visit (% warm, % cold)^d^	44.4%, 55.6%	44%, 56%
antibiotics (yes, no, % yes)	73, 107, 36%	11, 106, 9%
Infant Characteristics		
sex (male, female, % male)	95, 110, 46%	56, 66, 46%
delivery mode (vaginal, c-section, % c-section)	168, 37, 18%	89, 33, 27%
age (days)^e^	0–2	32.6 ± 3.3
birthweight (g)	3,050 ± 520.7	3,400 ± 400
birth length (cm)	47.9 ± 4.0	50.4 ± 2.4
gestational age at delivery^f^	24 (12%)	0 (0%)
preterm birth (between 22 and 0/7 and 36–6/7 weeks gestation)	71 (34%)	4 (3%)
early term (between 37 and 0/7 and 38–6/7 weeks gestation)	110 (54%)	115 (97%)
full term or late (≥39–0/7 weeks)		
breastfeedings per day^g^		6.6 ± 2.4
Traffic-related Air Pollution Exposure		
cumulative pregnancy NO_*x*_ (ppb)	73.9 ± 47.1	35.9 ± 7.6
1st trimester NO_*x*_ (ppb)	75.6 ± 44.1	35.8 ± 15.4
2nd trimester NO_*x*_ (ppb)	75.4 ± 48.6	36.0 ± 18.2
3rd trimester NO_*x*_ (ppb)	71.1 ± 54.1	36.1 ± 17.2

a In the Atlanta African American Maternal-Child Cohort, body mass index was calculated using measured height and self-reported prepregnancy weight during early pregnancy. In the Southern California Mother’s Milk Study, prepregnancy body mass index was assessed via questionnaire at the 1 month postpartum visit.

b The Southern California Mother’s Milk Study was limited to participants with no previous children.

c Potential participants in the Southern California Mother’s Milk Study were excluded if they reported any alcohol, tobacco, or recreational drug use during pregnancy.

d Study visits which occurred between April 1 and October 31 were considered warm season. All other visits were considered cold season.

e In the Atlanta African American Maternal-Child Cohort, metabolomics was measured using dried blood spots, which were collected within 48 h of delivery. In the Mother’s Milk Study, metabolomics was measured using fecal samples, collected at approximately 1 month postpartum.

f Potential participants were excluded from the Southern California Mother’s Milk Study if they had a preterm birth. Preterm, early term, and full or late term was categorized based on data collection in the Southern California Mother’s Milk Study and may differ from the American OBGYN definition of preterm birth.

g In the Atlanta African American Maternal-Child Cohort, breastfeeding was not included, as blood spot samples for metabolomics analysis were collected at birth, before breastfeeding would have begun.

### Prenatal NO_*x*_ Exposure was Associated with Level-1 Metabolites

Nonparametric PERMANOVA analysis was used to assess the associations between prenatal NO_*x*_ exposure and variance in the infant metabolome (Table S1). In the ATL AA Cohort, we found that prenatal NO_*x*_ exposure explained between 0.5 and 0.6% of variance in the HILIC chromatography column (*P*_all_ ≥ 0.1). For infants in the Mother’s Milk Study, we found that cumulative and second trimester NO_*x*_ exposure explained 2% of variance (*P* = 0.03 and 0.02, respectively) in the HILIC column and 2% of variance in the C18 column (*P* = 0.01 and 0.006, respectively).

We found that prenatal NO_*x*_ exposure was associated with the intensity of individual metabolites in each cohort (Table 2). A total of 8 [1] and 16 [11] Level-1 metabolites exhibited significant associations with prenatal NO_*x*_ exposure in ATL AA Cohort and Mother’s Milk Study, respectively (p value < 0.01 [*q* value < 0.2]). In ATL AA Cohort, several lipids, including nordeoxycholic acid, 7α-hydroxy-3-oxo-4-cholestenoic acid, and stearidonic acid, were significantly associated with prenatal NO_*x*_ exposure during the second and third trimesters, as well as across the entire pregnancy. Additionally, metabolites involved in xenobiotics metabolism were identified in every prenatal NO_*x*_ exposure window. In the Mother’s Milk Study, prenatal NO_*x*_ exposures were associated with a broader range of metabolites, including amino acids, carbohydrates, and lipids. Among these metabolites, perturbations in amino acids were found to be associated with all prenatal NO_*x*_ exposure windows. Specifically, most amino acids, including isoleucine, phenylalanine, valine/5-aminopentanoate, glutamic acid/methyl-aspartic acid, and histidine showed significant associations with prenatal NO_*x*_ exposure in the second trimester and throughout the entire pregnancy, and histidine was also significantly associated with NO_*x*_ exposure in the third trimester.

**Table 2:** Prenatal Exposure to NO_*x*_ during Each Trimester was Associated with Level-1 Metabolites^a^

Atlanta African American Maternal-Child Cohort (ATL AA)					Southern California Mother’s Milk Study (MMS)			
major pathway	metabolite	direction	*P*	*P*_BH_	metabolite	direction	*P*	*P*_BH_
cumulative pregnancy NO_x_								
HILIC								
amino acid					isoleucine	↓	0.001	**0.09**
					phenylalanine	↓	0.003	**0.12**
carbohydrate					glucosamine 6-phosphate	↑	0.00004	**0.006**
lipid	7α-hydroxy-3-oxo-4-cholestenoic acid	↑	0.01	0.44				
	nordeoxycholic acid	↓	0.01	0.30				
other	acetaminophen	↑	0.01	0.44	bufuralol	↑	0.003	**0.12**
	3,4,5-trimethoxybenzaldehyde	↓	0.0003	**0.07**				
	3,4-dihydroxybenzoic acid methyl ester	↓	0.01	0.44				
C18								
amino acid					valine/5-aminopentanoate	↓	0.001	**0.06**
					isoleucine/norleucine	↓	0.001	**0.06**
					glutamic acid/methyl-aspartic acid	↓	0.006	**0.16**
					histidine	↓	0.002	**0.06**

Atlanta African American Maternal-Child Cohort (ATL AA)					Southern California Mother’s Milk Study (MMS)			
major pathway	metabolite	direction	*P*	*P*_BH_	metabolite	direction	*P*	*P*_BH_
1st trimester								
HILIC								
amino acid					4-hydroxy-phenylglycine/pyridoxal	↓	0.001	**0.19**
lipids					sphinganine	↓	0.005	0.25
other	acetaminophen	↑	0.01	0.49	phosphocholine	↓	0.005	0.25
	carboxin	↓	0.01	0.49				
	3,4,5-trimethoxybenzaldehyde	↓	0.00008	**0.02**				
C18								
other					monoacylglycerol (14:0/0:0/0:0)	↑	0.004	0.39

Atlanta African American Maternal-Child Cohort (ATL AA)					Southern California Mother’s Milk Study (MMS)			
major pathway	metabolite	direction	*P*	*P*_BH_	metabolite	direction	*P*	*P*_BH_
2nd trimester								
HILIC								
amino acid					isoleucine	↓	0.0002	**0.02**
					asparagine	↓	0.007	**0.15**
					phenylalanine	↓	0.003	**0.10**
carbohydrate					nicotinamide (B3)	↓	0.009	**0.19**
					glucosamine 6-phosphate	↑	0.00001	**0.002**
lipid	7α-hydroxy-3-oxo-4-cholestenoic acid	↑	0.01	0.55				
other	3,4,5-trimethoxybenzaldehyde	↓	0.0002	**0.05**	serotonin/cotinine	↓	0.003	**0.10**
					alpha-tocopherol	↓	0.003	**0.10**
C18								
amino acid					valine/5-aminopentanoate	↓	0.01	0.32
					glutamic acid/methyl-aspartic acid	↓	0.005	0.32
					histidine	↓	0.007	0.32

Atlanta African American Maternal-Child Cohort (ATL AA)					Southern California Mother’s Milk Study (MMS)			
major pathway	metabolite	direction	*P*	*P*_BH_	metabolite	direction	*P*	*P*_BH_
3rd Trimester								
HILIC								
lipid	7α-hydroxy-3-oxo-4-cholestenoic acid	↑	0.01	0.30				
	nordeoxycholic acid	↓	0.01	0.30				
	stearidonic acid	↑	0.01	0.30				
other	acetaminophen	↑	0.01	0.30	phosphocholine	↑	0.009	0.56
	fenobucarb/promecarb	↑	0.01	0.30				
	3,4,5-trimethoxybenzaldehyde	↓	0.003	0.30				
	3,4-dihydroxybenzoic acid methyl ester	↓	0.003	0.30				
C18								
amino acid					histidine	↓	0.004	0.28
other					monoacylglycerol (14:0/0:0/0:0)	↓	0.006	0.28

a Results shown were generated using linear models where the outcome was the logged intensity of each metabolite, and the primary exposure was NO_*x*_. Models for the MMS were adjusted for infant age and sex, maternal education, maternal body mass index, maternal age, and season of study visit. Models for the ATL AA were additionally adjusted for alcohol, tobacco, and marijuana use during pregnancy. Results shown are those with *P*_raw_ < 0.01. **Bold** values indicate *P*_BH_ < 0.2.

### Prenatal NO_*x*_ Exposure was Associated with Perturbations in Metabolic Pathways Including Oxidative Stress and Inflammatory Response

To understand the associations between NO_*x*_ and the infant metabolome more holistically, we examined the associations between prenatal NO_*x*_ exposure and untargeted metabolomic features. The number of statistically significant findings at varying levels of statistical significance are summarized in Table S2. Briefly, we observed that the ATL AA Cohort had a greater number of statistically significant associations (*P*_BH_ < 0.2) with second trimester NO_*x*_ exposure. The MMS had a greater number of statistically significant associations (*P*_BH_ < 0.2) with cumulative and second trimester NO_*x*_ exposure. Based on these results, *Metapone* was used to identify putative metabolic pathways that were enriched in association with prenatal NO_*x*_ exposure, with local false discovery rate <0.2. In the ATL AA Cohort, cumulative prenatal NO_*x*_ exposure was associated with perturbations in eight metabolic pathways. Notably, only cytochrome p450 metabolism was found to be associated with first trimester NO_*x*_ exposure (Figure 2). In the MMS, selected pathways are shown in Figure 3. These pathways were chosen based on those that had statistically significant results across all prenatal exposure periods. Complete results for all pathways can be found in Table S3. Many of the pathways identified in Figure 3 are involved in oxidative stress and inflammatory response such as tyrosine metabolism, tryptophan metabolism, histidine metabolism, and pyrimidine metabolism. Pathways enriched in bile acid metabolism and nucleotide metabolism were uniquely identified in the MMS. In both cohorts, prenatal NO_*x*_ exposure was associated with enrichment in pathways related to methionine and cysteine metabolism, tryptophan metabolism, carbon metabolism, methane metabolism, glycerophospholipid metabolism, degradation of aromatic compounds, drug metabolism, cytochrome p450, and metabolism of xenobiotics by cytochrome p450.

**Figure 2:**
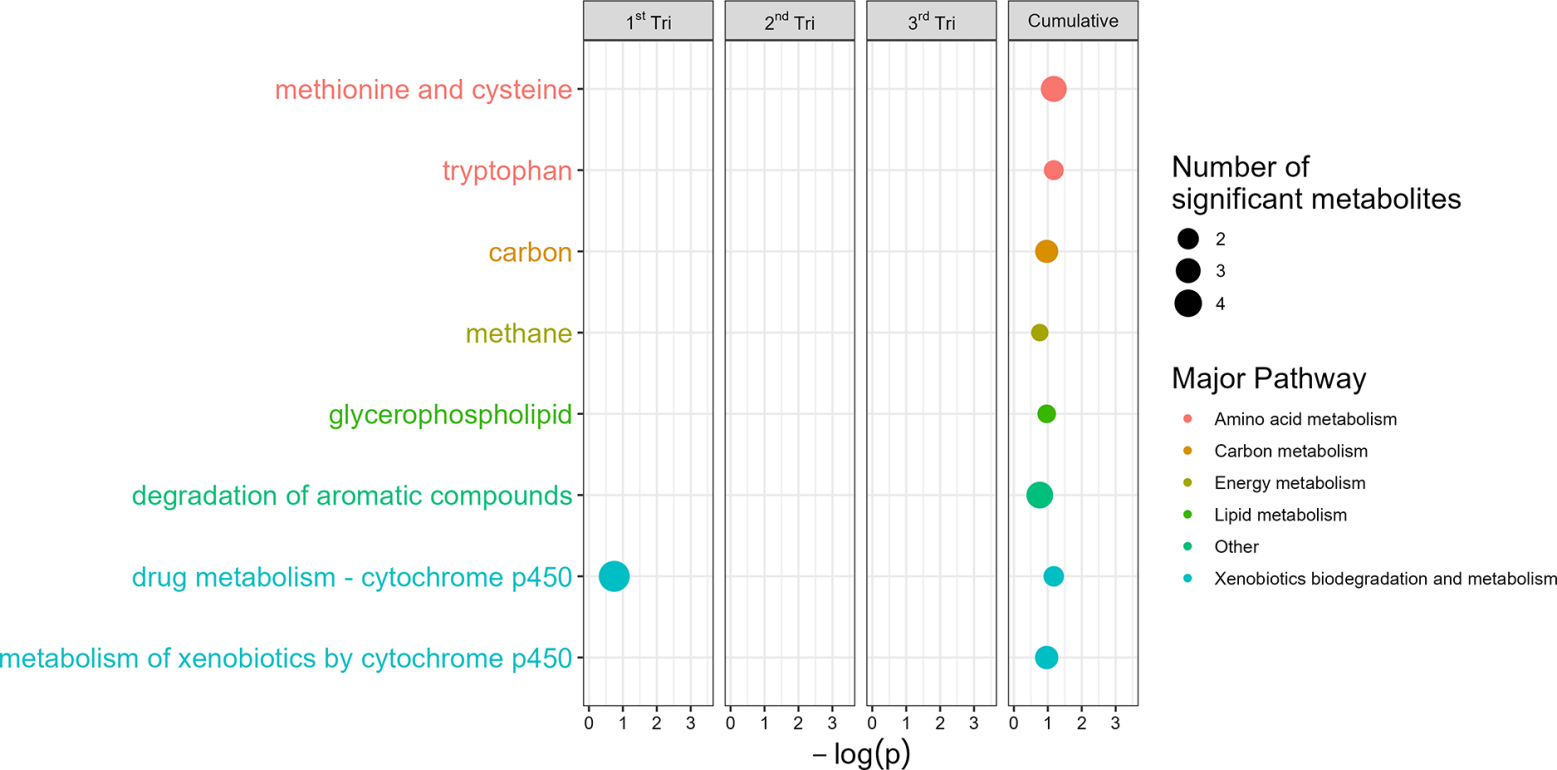
Prenatal NO_*x*_ exposure during pregnancy was associated with circulating metabolic pathways at birth in the Atlanta African American Maternal-Child Cohort. Pathways with local false discovery rate (lfdr) < 0.2 and number of significant metabolites in the pathway ≥2 were included. The *Y*-axis includes each metabolic pathway which met these criteria, and the *X*-axis represents the -log-transformed *p*-value of the association between air pollution exposure and each pathway. Relationships between NO_*x*_ exposure and untargeted metabolic features were estimated using linear models adjusted for infant age and infant sex, maternal education, season of study visit, maternal age, maternal prepregnancy body mass index, and alcohol, tobacco, and marijuana use during pregnancy.

**Figure 3:**
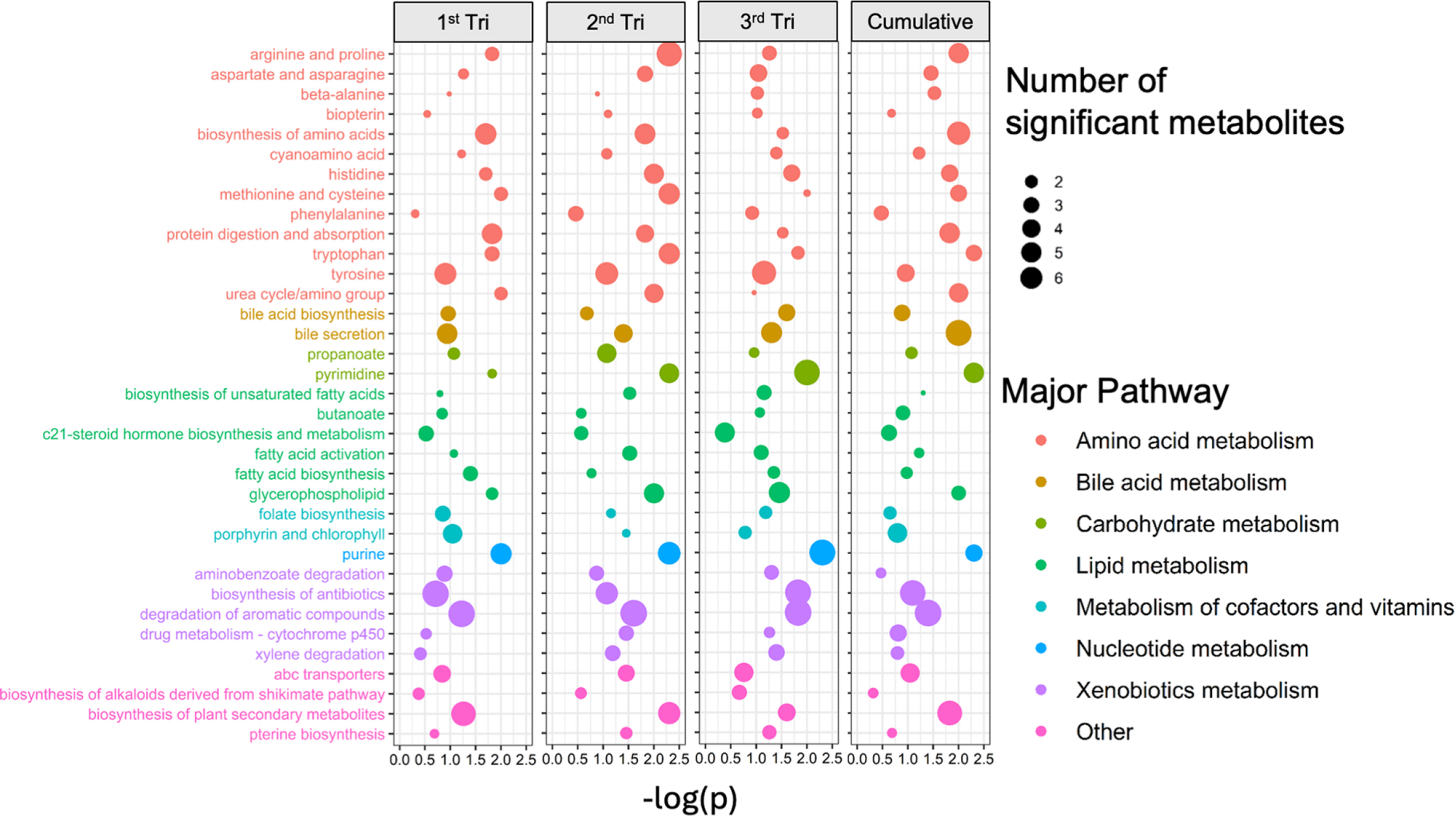
Prenatal NO_*x*_ exposure during pregnancy was associated with fecal metabolic pathways at 1 month of age in the Southern California Mother’s Milk Study. Pathways with lfdr (local false discovery rate) < 0.2 and number of significant metabolites in the pathway ≥2 were included. The *Y*-axis includes each metabolic pathway which met these criteria, and the *X*-axis represents the -log-transformed *p*-value of the association between air pollution exposure and each pathway. Relationships between NO_*x*_ exposure and untargeted metabolic features were estimated using linear models adjusted for infant age in days, infant sex, maternal education, season of study visit, mother age, and prepregnancy body mass index.

### Prenatal NO_*x*_ Exposure was Associated with Metabolic Features Detected in DBS and Fecal Samples

Overall, there were 1,125 metabolic features that were detected in both cohorts. Among these, several metabolic features were associated with prenatal NO_*x*_ exposure in both cohorts (Table S4). For example, in the ATL AA Cohort, when using a 20% false discovery rate (*P*_BH_ < 0.2), pregnancy exposure to NO_*x*_ was associated with five metabolic features, first trimester NO_*x*_ exposure with one metabolic feature, and second trimester NO_*x*_ exposure with two metabolic features. Similarly, in the MMS, cumulative pregnancy NO_*x*_ was associated with 12 metabolic features (*P*_BH_ < 0.2), second trimester NO_*x*_ with six features, and third trimester NO_*x*_ with seven metabolic features. With the results summarized in Table S4, *Metapone* was used to identify metabolic pathways that were enriched in association with prenatal NO_*x*_ exposure. In the ATL AA Cohort, prenatal NO_*x*_ exposures were consistently associated with tryptophan metabolism, glycerophospholipid metabolism, and degradation of aromatic compounds (Figure 4). Similarly, in the MMS, most of these pathways were also enriched in an independent analysis (Figure 5). Specifically, prenatal NO_*x*_ across all exposure windows was associated with fructose and mannose metabolism, anthocyanin metabolism, and retrograde endocannabinoid signaling. In both cohorts, prenatal NO_*x*_ exposure was associated with perturbations in pathways involved with glycerophospholipid metabolism, linoleate/linoleic acid, and degradation of aromatic compounds.

**Figure 4:**
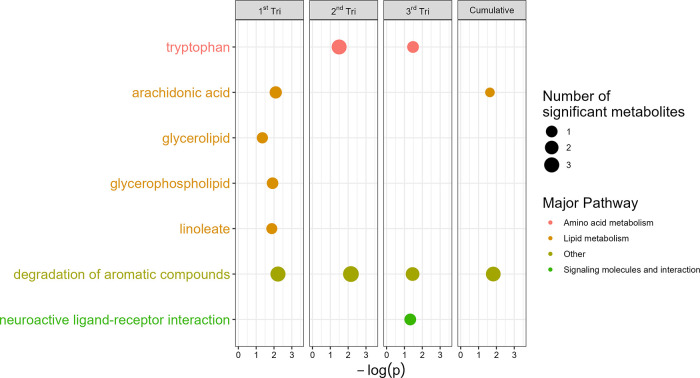
In the Atlanta African American Maternal-Child Cohort, prenatal NO_*x*_ exposure was associated with circulating metabolic pathways at birth, including only metabolites which were also identified in the Southern California Mother’s Milk Study at 1 month of age. Pathways with raw *p*-value <0.05 were included. The *Y*-axis includes each metabolic pathway which met these criteria, and the *X*-axis represents the -log-transformed p-value of the association between air pollution exposure and each pathway. Relationships between NO_*x*_ exposure and untargeted metabolic features were estimated using linear models adjusted for infant age and sex, maternal education, season of study visit, maternal age, maternal prepregnancy body mass index, and alcohol, tobacco, and marijuana use during pregnancy.

**Figure 5:**
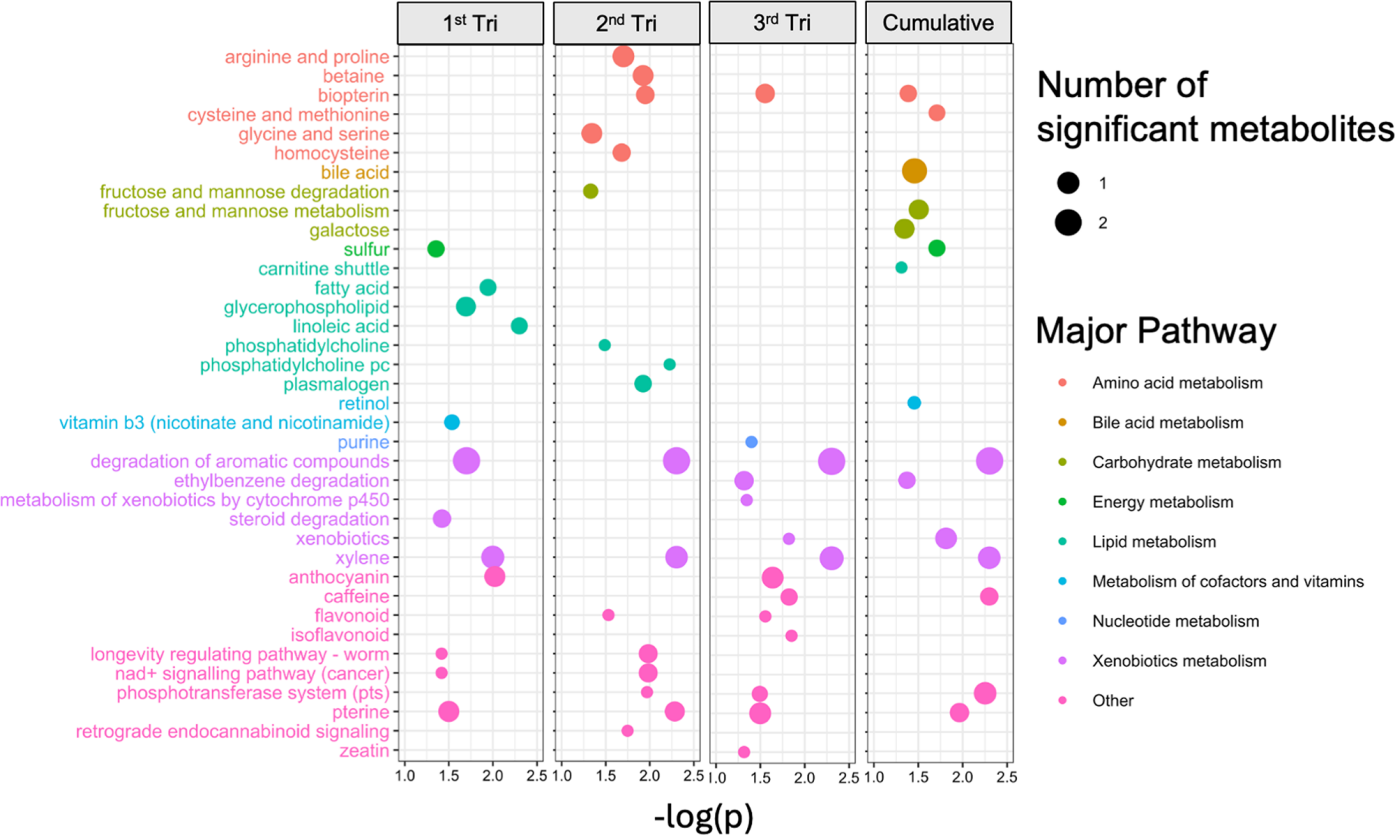
In the Southern California Mother’s Milk Study, prenatal NO_*x*_ exposure was associated with fecal metabolic pathways at 1 month of age, including only metabolites which were also identified in the Atlanta African American Maternal-Child Cohort at birth. Pathways with raw *p*-value <0.05 were included. The *Y*-axis includes each metabolic pathway which met these criteria, and the *X*-axis represents the -log-transformed *p*-value of the association between air pollution exposure and each pathway. Relationships between NO_*x*_ exposure and untargeted metabolic features were estimated using linear models adjusted for infant age and sex, maternal education, season of study visit, maternal age, and maternal prepregnancy body mass index.

## Discussion

To our knowledge, this is the first combined untargeted high-resolution metabolomics application conducted to investigate the association between prenatal TRAP exposure and the infant metabolome, encompassing two sample types from two minority populations from two geographic regions – Atlanta and Southern California. We found that prenatal NO_*x*_ exposures were linked with perturbations in pathways and metabolic signals involved in key biological processes, including antioxidant defense and neurotransmission (amino acid metabolism and neuroactive signaling metabolism), oxidative stress and inflammatory apoptosis (bile acid metabolism, metabolism of cofactors and vitamins), nucleic acid damage and repair (nucleotide metabolism), and energy homeostasis (carbohydrate metabolism, energy metabolism and lipid metabolism) within the newborn DBS and fecal metabolome. In exploring the cumulative pregnancy period, as well as each trimester, we have identified several metabolites whose intensity is consistently associated with NO_*x*_ exposure (i.e., 7α-hydroxy-3-oxo-4-cholestenoic acid and isoleucine). However, we also found that several metabolites were uniquely associated with air pollution exposure during only one trimester, highlighting the potential that exposures during each trimester may have distinct impacts, which may be expected due to observed differences in the effects of air pollution across different trimesters. For example, air pollution appears to have differing associations with fetal growth, depending on trimester of exposure.^45^ These findings highlight several potential biological mechanisms through which prenatal TRAP exposure may impact infant development.

### Our Findings were Largely Consistent with Previous Studies

Previous work in this area has assessed the association between TRAP and the metabolome in adults. For example, Cheng et al. assessed changes in serum metabolites before and after acute TRAP exposure and found perturbations in pathways related to fatty acid biosynthesis and tryptophan metabolism, among others.^21^ Similarly, found that prenatal TRAP exposure was associated with perturbation to tryptophan metabolism pathways in both cohorts and fatty acid metabolism in the MMS. Several previous studies have found that histidine is associated with TRAP exposure.^22–24^ Consistent with these previous studies, we found that TRAP exposure was inversely associated with histidine in the MMS. In one study which explored the associations between TRAP and DBS metabolites in infants, fatty acid metabolism and methionine and cysteine were associated with TRAP exposure.^25^ In concordance with this study, we found that methionine and cysteine metabolism pathways were perturbed in association with TRAP exposure in both cohorts and in the MMS, fatty acid activation and biosynthesis were perturbed in association with TRAP exposure.

### Common Perturbations in Amino Acid and Lipid Metabolism Across Two Cohorts

Prenatal NO_*x*_ exposures were associated with amino acid pathways, including tryptophan metabolism, methionine and cysteine metabolism, arginine and proline metabolism, aspartate and asparagine metabolism, histidine metabolism, tyrosine metabolism, glycine and serine metabolism, and the urea cycle/amino group. These findings align well with previous research linking prenatal TRAP exposures with alterations in amino acid pathways in maternal and infant metabolomes.^20,22,25,46,47^ Specifically, perturbation in tryptophan metabolism and methionine and cysteine metabolism were observed in both cohorts in the current study. Previous experimental and observational studies suggested that air pollution alters tryptophan metabolism.^23,48^ In addition to its antioxidant properties,^49^ tryptophan and its metabolites play a crucial role in supporting fetal growth and development,^50^ including brain serotonin synthesis in infants^51^ that can aid in neuronal development.^52^ Furthermore, air pollution has been associated with alterations in pathways of sulfur-containing amino acids, such as methionine, cysteine, and histidine, which are readily oxidized^53,54^ and are responsible for a majority of the reactive oxygen species (ROS) interactions with proteins.^55^ Animal studies have also described the oxidation of methionine in rodent lung and lung fluid exposed to PM_2.5_.^56,57^ Meanwhile, methionine and cysteine metabolism are vital for biological growth and redox balance.^58^ These findings suggest the potential adverse health effects of TRAP on infant development.

Prenatal NO_*x*_ exposures were also associated with perturbations in lipid and fatty acid metabolism in both cohorts, which are active inflammatory mediatory pathways. We identified several key pathways of lipid and fatty acid metabolisms (i.e., linoleate metabolism, glycerophospholipid metabolism, arachidonic acid metabolism, fatty acid activation), carnitine shuttle, butanoate metabolism (a pathway for short-chain fatty acids and alcohols), C21-steroid hormone biosynthesis and metabolism (synthesis of hormones such as glucocorticoids, androgens, estrogens, and progestogens), and unsaturated fatty acid biosynthesis. These metabolic perturbations were largely consistent with previous findings of TRAP exposure on pregnant women^20,22,25^ or other general populations.^59–61^ Among these pathways, linoleate metabolism, glycerophospholipid metabolism, and arachidonic acid metabolism were found to overlap between our two cohorts. A mouse study indicated that chronic exposure to TRAP can reduce levels of linoleic acid and arachidonic acid through inducing systemic inflammation.^62^ Additionally, NO_2_ and particulate matter can affect the methylation of certain genes, leading to changes in blood lipids and blood glucose level, thus affecting lipid metabolism.^63^ Linoleate and arachidonic acid metabolism, which belong to omega-6 polyunsaturated free fatty acid metabolism, have been shown to be activated by free radicals and oxidative stress generated from TRAP. This activation leads to the production of leukotrienes and prostaglandins, which are major proinflammatory mediators responsible for infant development.^64,65^

The metabolic feature annotation and validation findings in amino acid and lipid metabolism further agree with the pathway enrichment results. In the MMS, prenatal NO_*x*_ exposures were negatively associated with metabolic intensities of key amino acids, including isoleucine, phenylalanine, histidine, asparagine, and glutamic acid. These metabolites consistently exhibit significant associations with multiple air pollutants in several independent studies,^20^ and are closely linked with inflammatory responses and oxidative stress.^24,66–68^ Histidine and glutamic acid have been reported as antioxidants to reduce the susceptibility to oxidative stress and contribute to infant immune development.^69,70^ Thus, the reduction of these metabolites enriched for oxidative stress and inflammatory responses suggest that prenatal air pollution exposure may contribute to elevated oxidative stress that can be observed in infant fecal samples. Moreover, prenatal NO_*x*_ exposure was positively associated with sphinganine, one of the sphingolipids, in the MMS. Previous untargeted lipidomics revealed that short- and medium-term air pollution exposures were associated with sphingolipid metabolism.^71,72^ The perturbation of sphingolipids may contribute to cardiometabolic disease risk and air pollution-associated dysregulation of glucose metabolism.^73,74^ In the ATL AA Cohort, we found that greater exposure to prenatal NO_*x*_ was associated with increased levels of another lipid, stearidonic acid. This is consistent with a previous study where exposure to PM_2.5_ in mice caused an increase in stearidonic acid,^34^ which is an omega-3 polyunsaturated fatty acid that exerted antiinflammatory effects by suppressing nitric oxide synthase (NOS) mediated NO production.^75^

### Predominant Perturbations in Bile Acid, Carbohydrate, and Nucleotide Metabolism within the Fecal Metabolome

Our findings underscore the significant impact of prenatal NO_*x*_ exposure on the infant fecal metabolome, particularly in relation to bile acid, carbohydrate, and nucleotide metabolism, as observed in the MMS. In this cohort, prenatal NO_*x*_ exposures were associated with perturbations in these critical metabolic pathways, which are essential for gut microbiome development and infant health.^76^ In addition, the MMS revealed novel associations between prenatal NO_*x*_ exposure with other metabolites, including phosphocholine, glucosamine 6-phosphate, and nicotinamide (vitamin B3), which are involved in processes like membrane integrity, energy metabolism, and DNA repair. In the ATL AA Cohort, greater prenatal NO_*x*_ exposure was associated with metabolites in DBS that are key components of the primary bile acid biosynthesis pathway, including 7α-hydroxy-3-oxo-4-cholestenoic acid and nordeoxycholic acid. These findings suggest that prenatal TRAP exposure may lead to metabolic disruptions that influence gut microbiota development and overall infant health. Thus, the impact of TRAP on pathways critical for gut microbiome development was more prominently observed in the MMS. However, this may be due to the varying biomatrices used in each cohort.

### Implications of TRAP Exposure on Infant Neurodevelopment

The findings from both the ATL AA Cohort and the MMS reveal significant associations between prenatal NO_*x*_ exposures and metabolic pathways crucial for infant neurodevelopment. Notably, pathways such as neuroactive ligand–receptor interaction, retrograde endocannabinoid signaling, and tryptophan metabolism were disrupted in response to NO_*x*_ exposure across different trimesters and cumulatively throughout pregnancy. Tryptophan metabolism, a pathway involved in the synthesis of serotonin, a critical neurotransmitter,^77–79^ was associated with prenatal NO_*x*_ exposure. In the ATL AA Cohort, tryptophan metabolism was linked with prenatal NO_*x*_ exposure during the second and third trimesters, while in the MMS, the impact extended across all trimesters, highlighting a potentially critical window of vulnerability. Additionally, the neuroactive ligand–receptor interaction pathway, which is fundamental to neurotransmission and brain signaling, was associated with prenatal NO_*x*_ exposure. The persistent influence of TRAP on this pathway, particularly across multiple trimesters, suggests a sustained impact on neurodevelopmental processes. Prenatal NO_*x*_ exposure was also associated with retrograde endocannabinoid signaling, which is known to play a role in synaptic plasticity, neurogenesis, and the regulation of cognitive functions.^80–82^ The consistent perturbation of this pathway across all prenatal exposure windows in the MMS indicates that prenatal TRAP could interfere with the normal development of these critical neurodevelopmental processes, potentially leading to long-term cognitive and behavioral consequences.^83^

### Fecal and DBS Metabolomes Provide Insights into the Effects of Prenatal TRAP Exposure

The comparison between the fecal metabolome and the DBS metabolome provides critical insights into how different biological matrices capture the effects of prenatal exposures, such as TRAP, on infant metabolic profiles. The fecal metabolome captures metabolites that are influenced by both host and microbial processes. In our study, the fecal metabolome from the MMS revealed significant associations between prenatal NO_*x*_ exposure and pathways involved in bile acid metabolism, carbohydrate metabolism, and nucleotide metabolism. These pathways are essential for gut microbiome maturation and suggest that TRAP exposure may disrupt the development of the gut microbiota, potentially leading to long-term health implications. On the other hand, the DBS metabolome reflects systemic metabolic processes, including those related to lipid and amino acid metabolism, which are crucial for overall infant development. In the ATL AA Cohort, DBS samples revealed associations between prenatal NO_*x*_ exposures and metabolites involved in lipid metabolism, amino acid metabolism, and xenobiotic metabolism. These findings highlight the broader systemic effects of TRAP exposure, extending beyond the gut to influence various metabolic pathways that are vital for infant growth and immune function. Together, each of these matrices allows for a more holistic assessment of how prenatal exposures influence infant health, capturing both localized and systemic effects.

### Exogenous Metabolites and Xenobiotic Metabolism

Prenatal NO_*x*_ exposures in both cohorts were associated with xenobiotic pathways and metabolites, such as exogenous toxicants, industrial chemicals, pesticides, and pharmaceuticals, suggesting potential exposures to environmental pollutants and metabolic detoxification processes in early life. Specifically, five xenobiotic metabolites involved in aromatic compounds degradation were identified in the ATL AA Cohort. Consistently, all prenatal NO_*x*_ exposure windows were associated with degradation of aromatic compounds pathways. Among them, Carboxin, Fenobucarb and Promecarb, commonly used as pesticides, have been detected in human metabolome previously.^84^ The presence of these xenobiotic metabolites could indicate potential food contamination^85^ or residential pesticide exposure. These findings also highlight the need to consider coexposures to industrial and consumer product-related chemicals when assessing prenatal TRAP exposure. Importantly, we found 3,4,5-trimethoxybenzaldehyde, an intermediate in the synthesis of some pharmaceutical drugs, in all exposure windows, as well as acetaminophen in most of exposure windows in newborn DBS in ATL AA. We also found that acetaminophen was detected in over 95% of infants, suggesting widespread prenatal exposure. Given that acetaminophen is commonly used during pregnancy for fever and pain relief,^86^ its detection may reflect maternal medication use, passive transfer through the placenta, or neonatal administration shortly after birth. The presence of these exogenous drug-related metabolites, particularly acetaminophen, could have implications for the endogenous metabolic response in newborns. Acetaminophen metabolism involves glucuronidation and sulfation pathways, which are key components of xenobiotic detoxification processes.^87^ High levels of prenatal acetaminophen exposure could influence endogenous pathways related to oxidative stress, inflammation, and glutathione metabolism, potentially affecting the metabolic responses to environmental exposures such as NO_*x*_. Moreover, prior research suggests that prenatal acetaminophen exposure may alter metabolic homeostasis, including disruptions in lipid metabolism and inflammatory cytokine signaling, which could have long-term implications for immune development and neurodevelopmental outcomes.^88^ Meanwhile, the detection of other exogenous xenobiotic metabolites, including pesticide-related compounds and industry chemicals, suggests that maternal coexposures to environmental chemicals could further affect fetal metabolic responses. Future studies on exposomics to explore the role of coexposures and investigating potential links between air pollution exposures, pharmaceutical metabolism, and endogenous metabolic responses in early life are warranted.

### Study Limitations

This study provides insights into the effect of early life exposure to TRAP on metabolic pathways using different biomatrices. However, it is important to acknowledge the limitations that may have impacted our findings, including the absence of data on indoor nitrogen oxides, which are significant sources of exposure, especially in environments where gas stoves and heaters are used without proper ventilation.^89^ Additionally, while potential participants were excluded from the MMS if they reported smoking, we were not able to assess the impact of exposure to environmental tobacco smoke in this cohort. Future studies should consider these indoor sources. Additionally, this study analyzed NO_*x*_ levels as an estimate of TRAP exposure. There are several other traffic-related pollutants which may also have important implications for the infant metabolome, including volatile organic compounds, ozone, and fine particles. Furthermore, the two cohorts had differing exposure to NOx, with the having a cumulative mean exposure of 74 ppb compared to 36 ppb in the MSS. We are also limited in our ability to directly compare levels of NOx across cohorts, since TRAP is a complex mixture with variable composition depending on traffic density and meteorological conditions. The lack of metabolomics profiling via the C18 column in the ATL AA Cohort also limited our ability to assess potential overlap of metabolite features detected by this method. In this study, we used a false discovery rate threshold of 20% to balance the need to identify significant associations while controlling for the risk of false positives. In analyses restricted to only metabolites observed in both cohorts, we used a threshold of *P* < 0.05. These relatively lenient thresholds were chosen to capture a broader range of metabolic features that may be affected by prenatal NO_*x*_ exposures. While this approach increases the likelihood of identifying true associations, it means that some of the findings may require validation in future studies. However, the primary goal of this study, and particularly the pathway analysis, is hypothesis generation and pathway analyses are tolerant to errors at an individual feature level.^90^ Although the use of different biometric samples allowed us to investigate associations between NO_*x*_ and infant metabolomics across sample types, the use of different samples for metabolic profiling introduces variability that may impact the comparability of results across studies. Therefore, this study represents a preliminary comparison across biological matrices but cannot be considered a validation or replication study. Standardizing the types of samples used could improve the consistency and generalizability of findings. Lastly, our study was limited by the scope of the metabolome analyses. The DBS metabolome was restricted to human pathways, whereas the fecal metabolome includes both human and microbiome-related pathways, potentially resulting in a broader range of pathways identified in the latter. Future studies should strive to integrate both human and microbiome pathways across different biomatrices to gain a more holistic understanding of metabolic processes.

## Supplementary Material



## Notes

Table S1. Results of PERMANOVA model assessing the relationship between prenatal NO_*x*_ exposure and Level-1 Metabolites. Table S2. Number of statistically significant associations between prenatal NO_*x*_ exposure and metabolite intensity at various significance thresholds, including all untargeted metabolites measured in each cohort. Table S3. Complete Metapone results assessing the association between prenatal NO*x* exposure and fecal metabolic pathways at 1 month of age in the Mother’s Milk Study. Table S4. Number of statistically significant associations between prenatal NO_*x*_ exposure and metabolite intensity at various significance thresholds, including only untargeted metabolites which were identified in both cohorts (PDF)
