# Supplementary material for: Prenatal Nitrogen Oxide (NO x ) and Its Potential Impact on Infant Metabolism during the First Month of Life: Evidence from Two Distinct Cohorts—The Atlanta African American Maternal-Child Cohort and the Southern California Mother’s Milk Study
Source: Environ Sci Technol. 2025 Sep 4;59(36):19131–45. doi: 10.1021/acs.est.5c04955 (PMC12444982; doi:10.1021/acs.est.5c04955)
Supplement: Supplementary file 1 [file es5c04955_si_001.pdf]

**Prenatal Nitrogen Oxide ($NO_x$) and its Potential Impact on Infant Metabolism during the First Month of Life: Evidence from Two Distinct Cohorts – the Atlanta African American Maternal-Child Cohort and the Southern California Mother's Milk Study**

Elizabeth A Holzhausen[1#], Youran Tan[2#], Nathan Young[1], Roshonda B Jones[1], Ziyin Tang[2], Jeremy A Sarnat[2], Fredrick Lurmann[3], Howard H Chang[2], ViLinh Tran[4], Dean P Jones[4], Michael I Goran[5], Anne L Dunlop[4], Donghai Liang[2,+,*], Tanya L Alderete[1,+,*]

**Supporting Information** (4 tables, 10 pages)

**Supplemental Table 1.** Results of PERMANOVA model assessing the relationship between prenatal $NO_x$ exposure and Level-1 Metabolites.

| Exposure | Atlanta African American Maternal-Child Cohort | | Mother's Milk Study | |
|---|---|---|---|---|
| | $R^2$ | P | $R^2$ | P |
| **HILIC Chromatography Column** | | | | |
| Cumulative Pregnancy $NO_x$ | 0.006 | 0.18 | 0.02 | **0.03** |
| 1st trimester $NO_x$ | 0.005 | 0.40 | 0.01 | 0.16 |
| 2nd trimester $NO_x$ | 0.006 | 0.10 | 0.02 | **0.02** |
| 3rd trimester $NO_x$ | 0.006 | 0.11 | 0.01 | 0.06 |
| **C18 Chromatography Column** | | | | |
| Cumulative Pregnancy $NO_x$ | - | - | 0.02 | **0.01** |
| 1st trimester $NO_x$ | - | - | 0.01 | 0.17 |
| 2nd trimester $NO_x$ | - | - | 0.02 | **0.006** |
| 3rd trimester $NO_x$ | - | - | 0.01 | 0.12 |

**Supplemental Table 1.** Unadjusted PERMANOVA models used Euclidian distance to estimate the association between overall metabolomics profiles and $NO_x$ exposure. **Bold** values indicate statistical significance at $P < 0.05$. $R^2$ and p-values are not reported for the C18 column in the ATL AA cohort due to the lack of metabolomics data.

**Supplemental Table 2.** Number of statistically significant associations between prenatal $NO_x$ exposure and metabolite intensity at various significance thresholds, including all untargeted metabolites measured in each cohort.

| | Atlanta African American Maternal-Child Cohort | | | | | | Mother's Milk Study | | | | | |
|---|---|---|---|---|---|---|---|---|---|---|---|---|
| **Exposure** | **P<0.05** | **P<0.01** | **P<0.005** | **P<0.0005** | **$P_{BH}$<0.20** | **$P_{BH}$<0.05** | **P<0.05** | **P<0.01** | **P<0.005** | **P<0.0005** | **$P_{BH}$<0.20** | **$P_{BH}$<0.05** |
| **Cumulative Pregnancy $NO_x$** | 512 | 126 | 66 | 7 | 0 | 0 | 1125 | 271 | 150 | 26 | 12 | 1 |
| **1st Trimester $NO_x$** | 500 | 99 | 55 | 3 | 0 | 0 | 1048 | 222 | 124 | 12 | 2 | 0 |
| **2nd Trimester $NO_x$** | 360 | 85 | 50 | 5 | 2 | 1 | 1148 | 247 | 152 | 27 | 12 | 0 |
| **3rd Trimester $NO_x$** | 344 | 70 | 35 | 3 | 0 | 0 | 1091 | 232 | 127 | 24 | 6 | 4 |

**Supplemental Table 2.** Results were generated using linear models, where the outcome was logged metabolite intensity. Models for the Mother's Milk Study were adjusted for infant age and sex, maternal education, maternal body mass index, maternal age, and season of study visit. Models for the Atlanta African American Maternal-Child cohort were additionally adjusted for alcohol, tobacco, and marijuana use during pregnancy.

**Supplemental Table 3.** Complete Metapone results assessing the association between prenatal $NO_x$ exposure and fecal metabolic pathways at 1-month of age in the Mother's Milk Study.

| Pathway | Cumulative Pregnancy | | | 1st Trimester | | | 2nd Trimester | | | 3rd Trimester | | |
|---|---|---|---|---|---|---|---|---|---|---|---|---|
| | Number significant metabolites | lfdr | P | Number significant metabolites | lfdr | P | Number significant metabolites | lfdr | P | Number significant metabolites | lfdr | P |
| 2-oxocarboxylic acid metabolism | 3.4 | 0.1 | 0.0 | 2.8 | 0.1 | 0.0 | 3.7 | 0.1 | 0.0 | NA | NA | NA |
| abc transporters | 3.5 | 0.2 | 0.1 | 2.6 | 0.1 | 0.1 | 3.1 | 0.1 | 0.0 | 2.8 | 0.1 | 0.2 |
| alanine and aspartate metabolism | 1.4 | 0.1 | 0.0 | NA | NA | NA | 2.4 | 0.1 | 0.0 | 1.3 | 0.1 | 0.1 |
| alanine metabolism | NA | NA | NA | NA | NA | NA | 1.8 | 0.1 | 0.1 | NA | NA | NA |
| alpha linolenic acid and linoleic acid metabolism | NA | NA | NA | NA | NA | NA | NA | NA | NA | 1.2 | 0.1 | 0.2 |
| alpha-linolenic acid metabolism | NA | NA | NA | NA | NA | NA | NA | NA | NA | 1.3 | 0.1 | 0.2 |
| amino sugar and nucleotide sugar metabolism | NA | NA | NA | NA | NA | NA | NA | NA | NA | 1.3 | 0.1 | 0.2 |
| amino sugar metabolism | NA | NA | NA | NA | NA | NA | 2.5 | 0.1 | 0.0 | 1.1 | 0.1 | 0.1 |
| aminoacyl-trna biosynthesis | 2.8 | 0.1 | 0.0 | 1.7 | 0.1 | 0.1 | 1.9 | 0.1 | 0.1 | NA | NA | NA |
| aminobenzoate degradation | 1.6 | 0.2 | 0.3 | 2.3 | 0.1 | 0.1 | 2.5 | 0.1 | 0.1 | 1.9 | 0.1 | 0.0 |
| ammonia recycling | NA | NA | NA | NA | NA | NA | 2.5 | 0.1 | 0.1 | NA | NA | NA |
| androgen and estrogen metabolism | NA | NA | NA | 1.4 | 0.1 | 0.4 | 1.1 | 0.1 | 0.5 | 1.1 | 0.2 | 0.7 |
| anthocyanin biosynthesis | NA | NA | NA | NA | NA | NA | NA | NA | NA | 1.5 | 0.1 | 0.1 |
| arachidonic acid metabolism | NA | NA | NA | 1.8 | 0.1 | 0.4 | NA | NA | NA | NA | NA | NA |
| arginine and proline metabolism | 3.9 | 0.1 | 0.0 | 1.9 | 0.1 | 0.0 | 6.6 | 0.0 | 0.0 | 1.9 | 0.1 | 0.1 |
| arginine biosynthesis | 1.4 | 0.2 | 0.2 | NA | NA | NA | NA | NA | NA | NA | NA | NA |
| ascorbate (vitamin c) and aldarate metabolism | NA | NA | NA | NA | NA | NA | 1.7 | 0.1 | 0.3 | 1.2 | 0.1 | 0.2 |
| ascorbate and aldarate metabolism | NA | NA | NA | NA | NA | NA | 1.1 | 0.1 | 0.3 | 1.2 | 0.1 | 0.2 |
| aspartate and asparagine metabolism | 2.4 | 0.1 | 0.0 | 1.4 | 0.1 | 0.1 | 2.8 | 0.1 | 0.0 | 2.4 | 0.1 | 0.1 |
| aspartate metabolism | NA | NA | NA | NA | NA | NA | 1.6 | 0.1 | 0.0 | 1.3 | 0.1 | 0.3 |
| benzoate degradation | 2.5 | 0.2 | 0.2 | NA | NA | NA | 2.6 | 0.1 | 0.2 | NA | NA | NA |

| | | | | | | | | | | | | |
|---|---|---|---|---|---|---|---|---|---|---|---|---|
| beta-alanine metabolism | 2.1 | 0.1 | 0.0 | 1.1 | 0.1 | 0.1 | 1.2 | 0.1 | 0.1 | 1.7 | 0.1 | 0.1 |
| betaine metabolism | 1.6 | 0.1 | 0.0 | NA | NA | NA | 2.6 | 0.1 | 0.1 | NA | NA | NA |
| bile acid biosynthesis | 2.7 | 0.2 | 0.1 | 2.1 | 0.1 | 0.1 | 2.2 | 0.1 | 0.2 | 2.4 | 0.1 | 0.0 |
| bile secretion | 6.2 | 0.1 | 0.0 | 3.3 | 0.1 | 0.1 | 3.6 | 0.1 | 0.0 | 3.2 | 0.1 | 0.0 |
| biopterin metabolism | 1.3 | 0.2 | 0.2 | 1.1 | 0.1 | 0.3 | 1.3 | 0.1 | 0.1 | 1.4 | 0.1 | 0.1 |
| biosynthesis of alkaloids derived from histidine and purine | 2.1 | 0.2 | 0.2 | 1.9 | 0.1 | 0.2 | 1.8 | 0.1 | 0.2 | NA | NA | NA |
| biosynthesis of alkaloids derived from ornithine, lysine and nicotinic acid | 2.6 | 0.2 | 0.4 | NA | NA | NA | 2.2 | 0.1 | 0.3 | NA | NA | NA |
| biosynthesis of alkaloids derived from shikimate pathway | 1.5 | 0.2 | 0.5 | 1.6 | 0.1 | 0.4 | 1.8 | 0.1 | 0.3 | 2.0 | 0.1 | 0.2 |
| biosynthesis of amino acids | 5.0 | 0.1 | 0.0 | 3.6 | 0.1 | 0.0 | 4.5 | 0.1 | 0.0 | 1.6 | 0.1 | 0.0 |
| biosynthesis of antibiotics | 6.1 | 0.2 | 0.1 | 5.4 | 0.1 | 0.2 | 5.2 | 0.1 | 0.1 | 4.7 | 0.1 | 0.0 |
| biosynthesis of phenylpropanoids | NA | NA | NA | NA | NA | NA | NA | NA | NA | 1.0 | 0.1 | 0.4 |
| biosynthesis of plant hormones | NA | NA | NA | 1.0 | 0.1 | 0.4 | 1.5 | 0.1 | 0.3 | NA | NA | NA |
| biosynthesis of plant secondary metabolites | 5.7 | 0.1 | 0.0 | 4.7 | 0.1 | 0.1 | 5.1 | 0.0 | 0.0 | 2.5 | 0.1 | 0.0 |
| biosynthesis of unsaturated fatty acids | 1.2 | 0.2 | 0.0 | 1.1 | 0.1 | 0.2 | 2.0 | 0.1 | 0.0 | 2.0 | 0.1 | 0.1 |
| butanoate metabolism | 2.3 | 0.2 | 0.1 | 1.5 | 0.1 | 0.1 | 1.6 | 0.1 | 0.3 | 1.4 | 0.1 | 0.1 |
| butyrate metabolism | NA | NA | NA | NA | NA | NA | 1.2 | 0.1 | 0.5 | NA | NA | NA |
| c21-steroid hormone biosynthesis and metabolism | 2.6 | 0.2 | 0.2 | 2.2 | 0.1 | 0.3 | 2.3 | 0.1 | 0.3 | 3.0 | 0.1 | 0.4 |
| c5-branched dibasic acid metabolism | NA | NA | NA | 1.1 | 0.1 | 0.1 | NA | NA | NA | NA | NA | NA |
| caffeine metabolism | 3.7 | 0.1 | 0.0 | 2.8 | 0.1 | 0.0 | 3.1 | 0.0 | 0.0 | NA | NA | NA |
| caprolactam degradation | NA | NA | NA | 1.1 | 0.1 | 0.1 | NA | NA | NA | NA | NA | NA |
| carbon metabolism | 2.1 | 0.2 | 0.1 | 2.2 | 0.1 | 0.3 | 1.9 | 0.1 | 0.0 | NA | NA | NA |
| cardiolipin biosynthesis cl | NA | NA | NA | NA | NA | NA | 1.2 | 0.1 | 0.2 | NA | NA | NA |
| carnitine shuttle | 1.2 | 0.2 | 0.2 | NA | NA | NA | 1.1 | 0.1 | 0.2 | 1.1 | 0.1 | 0.1 |
| catecholamine biosynthesis | NA | NA | NA | 1.1 | 0.1 | 0.2 | 1.1 | 0.1 | 0.2 | NA | NA | NA |
| central carbon metabolism in cancer | 2.8 | 0.1 | 0.0 | 1.9 | 0.1 | 0.1 | 2.1 | 0.1 | 0.1 | NA | NA | NA |
| chemical carcinogenesis | 2.2 | 0.2 | 0.1 | 1.1 | 0.1 | 0.1 | 2.4 | 0.1 | 0.0 | NA | NA | NA |

| | | | | | | | | | | | | |
|---|---|---|---|---|---|---|---|---|---|---|---|---|
| chloroalkane and chloroalkene degradation | NA | NA | NA | NA | NA | NA | 1.5 | 0.1 | 0.0 | NA | NA | NA |
| chlorocyclohexane and chlorobenzene degradation | 1.6 | 0.2 | 0.1 | NA | NA | NA | NA | NA | NA | NA | NA | NA |
| citric acid cycle | NA | NA | NA | NA | NA | NA | NA | NA | NA | 1.3 | 0.1 | 0.2 |
| cyanoamino acid metabolism | 1.9 | 0.2 | 0.1 | 1.2 | 0.1 | 0.1 | 1.7 | 0.1 | 0.1 | 1.6 | 0.1 | 0.0 |
| cysteine and methionine metabolism | 1.5 | 0.1 | 0.0 | 1.2 | 0.1 | 0.0 | 1.8 | 0.0 | 0.0 | NA | NA | NA |
| cysteine metabolism | NA | NA | NA | NA | NA | NA | 1.4 | 0.1 | 0.1 | NA | NA | NA |
| d-arginine and d-ornithine metabolism | 1.9 | 0.2 | 0.1 | NA | NA | NA | NA | NA | NA | NA | NA | NA |
| de novo fatty acid biosynthesis | 1.1 | 0.2 | 0.1 | NA | NA | NA | 1.5 | 0.1 | 0.2 | 1.3 | 0.1 | 0.1 |
| degradation of aromatic compounds | 6.5 | 0.2 | 0.0 | 5.5 | 0.1 | 0.1 | 7.3 | 0.1 | 0.0 | 5.0 | 0.1 | 0.0 |
| drug metabolism - cytochrome p450 | 2.9 | 0.2 | 0.2 | 1.4 | 0.1 | 0.3 | 2.6 | 0.1 | 0.0 | 1.5 | 0.1 | 0.1 |
| drug metabolism - other enzymes | NA | NA | NA | 2.3 | 0.1 | 0.0 | NA | NA | NA | 1.3 | 0.1 | 0.0 |
| ethanol degradation | NA | NA | NA | NA | NA | NA | 1.4 | 0.1 | 0.4 | NA | NA | NA |
| ethylbenzene degradation | 1.5 | 0.2 | 0.1 | NA | NA | NA | 2.1 | 0.1 | 0.1 | NA | NA | NA |
| fatty acid activation | 1.5 | 0.2 | 0.1 | 1.2 | 0.1 | 0.1 | 2.5 | 0.1 | 0.0 | 2.0 | 0.1 | 0.1 |
| fatty acid biosynthesis | 1.8 | 0.2 | 0.1 | 2.0 | 0.1 | 0.0 | 1.5 | 0.1 | 0.2 | 1.6 | 0.1 | 0.0 |
| fatty acid metabolism | 1.0 | 0.2 | 0.2 | NA | NA | NA | 2.7 | 0.1 | 0.1 | 2.1 | 0.1 | 0.3 |
| folate biosynthesis | 2.0 | 0.2 | 0.2 | 2.2 | 0.1 | 0.1 | 1.5 | 0.1 | 0.1 | 1.7 | 0.1 | 0.1 |
| fructose and mannose degradation | NA | NA | NA | NA | NA | NA | 1.1 | 0.1 | 0.2 | NA | NA | NA |
| fructose and mannose metabolism | NA | NA | NA | NA | NA | NA | NA | NA | NA | 1.0 | 0.1 | 0.1 |
| galactose metabolism | NA | NA | NA | 1.0 | 0.1 | 0.4 | NA | NA | NA | NA | NA | NA |
| glucosinolate biosynthesis | 1.6 | 0.2 | 0.1 | 1.3 | 0.1 | 0.1 | 1.8 | 0.1 | 0.1 | NA | NA | NA |
| glutamate metabolism | NA | NA | NA | NA | NA | NA | 1.7 | 0.1 | 0.1 | NA | NA | NA |
| glutathione metabolism | 1.5 | 0.2 | 0.2 | NA | NA | NA | NA | NA | NA | NA | NA | NA |
| glycerophospholipid metabolism | 2.3 | 0.1 | 0.0 | 1.6 | 0.1 | 0.0 | 4.2 | 0.0 | 0.0 | 3.3 | 0.1 | 0.0 |
| glycine and serine metabolism | 2.7 | 0.2 | 0.0 | 1.0 | 0.1 | 0.1 | 4.3 | 0.1 | 0.1 | NA | NA | NA |
| glycine, serine and threonine metabolism | 1.1 | 0.2 | 0.0 | 1.0 | 0.1 | 0.1 | 2.3 | 0.1 | 0.0 | NA | NA | NA |

| | | | | | | | | | | | | |
|---|---|---|---|---|---|---|---|---|---|---|---|---|
| glycine, serine, alanine and threonine metabolism | 3.0 | 0.1 | 0.0 | 1.4 | 0.1 | 0.1 | 5.2 | 0.1 | 0.0 | NA | NA | NA |
| glycolysis and gluconeogenesis | NA | NA | NA | NA | NA | NA | NA | NA | NA | 1.3 | 0.1 | 0.1 |
| histidine metabolism | 3.0 | 0.1 | 0.0 | 1.8 | 0.1 | 0.0 | 4.1 | 0.0 | 0.0 | 2.3 | 0.1 | 0.0 |
| inositol metabolism | NA | NA | NA | 2.8 | 0.1 | 0.2 | 1.2 | 0.1 | 0.2 | NA | NA | NA |
| inositol phosphate metabolism | NA | NA | NA | 2.8 | 0.1 | 0.3 | NA | NA | NA | NA | NA | NA |
| intracellular signalling through adenosine receptor a2a and adenosine | NA | NA | NA | NA | NA | NA | 1.2 | 0.1 | 0.2 | NA | NA | NA |
| linoleate metabolism | NA | NA | NA | NA | NA | NA | NA | NA | NA | 1.5 | 0.1 | 0.1 |
| linoleic acid metabolism | NA | NA | NA | 1.1 | 0.1 | 0.0 | NA | NA | NA | 1.4 | 0.1 | 0.1 |
| lysine degradation | NA | NA | NA | 1.4 | 0.1 | 0.1 | 1.1 | 0.1 | 0.1 | NA | NA | NA |
| metabolism of xenobiotics by cytochrome p450 | 3.4 | 0.1 | 0.0 | 1.5 | 0.1 | 0.1 | 2.4 | 0.1 | 0.0 | NA | NA | NA |
| methane metabolism | 2.0 | 0.2 | 0.1 | 2.1 | 0.1 | 0.2 | 1.8 | 0.1 | 0.1 | NA | NA | NA |
| methionine and cysteine metabolism | 2.8 | 0.1 | 0.0 | 1.8 | 0.1 | 0.0 | 4.7 | 0.0 | 0.0 | 1.2 | 0.1 | 0.0 |
| methionine metabolism | 1.7 | 0.2 | 0.1 | NA | NA | NA | 2.6 | 0.1 | 0.1 | NA | NA | NA |
| mineral absorption | 1.3 | 0.2 | 0.3 | NA | NA | NA | 1.0 | 0.1 | 0.2 | NA | NA | NA |
| mitochondrial beta-oxidation of long chain saturated fatty acids | NA | NA | NA | NA | NA | NA | 1.3 | 0.1 | 0.2 | NA | NA | NA |
| mitochondrial beta-oxidation of short chain saturated fatty acids | NA | NA | NA | NA | NA | NA | 1.2 | 0.1 | 0.4 | NA | NA | NA |
| monobactam biosynthesis | NA | NA | NA | NA | NA | NA | 1.1 | 0.1 | 0.1 | NA | NA | NA |
| neuroactive ligand-receptor interaction | NA | NA | NA | 1.2 | 0.1 | 0.0 | 1.3 | 0.1 | 0.2 | NA | NA | NA |
| nicotinate and nicotinamide metabolism | 1.2 | 0.1 | 0.0 | NA | NA | NA | 3.4 | 0.1 | 0.0 | NA | NA | NA |
| pantothenate and coa biosynthesis | 1.5 | 0.1 | 0.0 | 1.3 | 0.1 | 0.0 | 1.9 | 0.1 | 0.1 | NA | NA | NA |
| parkinson's disease | NA | NA | NA | NA | NA | NA | 1.9 | 0.1 | 0.2 | NA | NA | NA |
| pentose and glucuronate interconversions | NA | NA | NA | NA | NA | NA | 2.5 | 0.1 | 0.0 | NA | NA | NA |
| phenylalanine and tyrosine metabolism | NA | NA | NA | NA | NA | NA | 1.5 | 0.1 | 0.3 | NA | NA | NA |
| phenylalanine metabolism | 2.4 | 0.2 | 0.3 | 1.2 | 0.1 | 0.5 | 2.7 | 0.1 | 0.3 | 1.8 | 0.1 | 0.1 |
| phenylpropanoid biosynthesis | 1.3 | 0.2 | 0.4 | NA | NA | NA | NA | NA | NA | NA | NA | NA |
| phosphatidylcholine biosynthesis | NA | NA | NA | NA | NA | NA | 1.7 | 0.1 | 0.0 | NA | NA | NA |

| | | | | | | | | | | | | |
|---|---|---|---|---|---|---|---|---|---|---|---|---|
| phosphatidylcholine biosynthesis pc | NA | NA | NA | NA | NA | NA | 1.7 | 0.1 | 0.0 | NA | NA | NA |
| phosphatidylinositol phosphate metabolism | 1.0 | 0.2 | 0.3 | 3.1 | 0.1 | 0.0 | NA | NA | NA | NA | NA | NA |
| phosphatidylinositol signaling system | NA | NA | NA | 2.3 | 0.1 | 0.0 | NA | NA | NA | 1.5 | 0.1 | 0.1 |
| phosphonate and phosphinate metabolism | NA | NA | NA | NA | NA | NA | 1.6 | 0.1 | 0.1 | NA | NA | NA |
| phosphotransferase system (pts) | 1.3 | 0.2 | 0.4 | NA | NA | NA | NA | NA | NA | NA | NA | NA |
| phytanic acid peroxisomal oxidation | NA | NA | NA | NA | NA | NA | 1.2 | 0.1 | 0.4 | 1.0 | 0.1 | 0.4 |
| plasmalogen synthesis | NA | NA | NA | NA | NA | NA | 1.6 | 0.1 | 0.1 | NA | NA | NA |
| porphyrin and chlorophyll metabolism | 3.6 | 0.2 | 0.2 | 3.1 | 0.1 | 0.1 | 1.4 | 0.1 | 0.0 | 1.7 | 0.1 | 0.2 |
| porphyrin metabolism | 2.2 | 0.2 | 0.3 | 2.4 | 0.1 | 0.3 | 1.4 | 0.1 | 0.3 | NA | NA | NA |
| propanoate metabolism | 1.8 | 0.2 | 0.1 | 1.6 | 0.1 | 0.1 | 3.9 | 0.1 | 0.1 | 1.4 | 0.1 | 0.1 |
| protein digestion and absorption | 4.0 | 0.1 | 0.0 | 3.4 | 0.1 | 0.0 | 3.4 | 0.1 | 0.0 | 1.5 | 0.1 | 0.0 |
| pterine biosynthesis | 1.5 | 0.2 | 0.2 | 1.3 | 0.1 | 0.2 | 1.9 | 0.1 | 0.0 | 1.8 | 0.1 | 0.1 |
| purine metabolism | 2.9 | 0.1 | 0.0 | 3.6 | 0.1 | 0.0 | 5.3 | 0.0 | 0.0 | 4.7 | 0.1 | 0.0 |
| pyrimidine metabolism | 3.9 | 0.1 | 0.0 | 1.3 | 0.1 | 0.0 | 4.1 | 0.0 | 0.0 | 4.6 | 0.1 | 0.0 |
| pyruvate metabolism | NA | NA | NA | NA | NA | NA | 3.4 | 0.1 | 0.0 | 1.3 | 0.1 | 0.2 |
| riboflavin metabolism | NA | NA | NA | NA | NA | NA | 1.6 | 0.1 | 0.3 | 1.1 | 0.1 | 0.2 |
| secondary bile acid biosynthesis | 1.1 | 0.2 | 0.3 | NA | NA | NA | NA | NA | NA | 1.5 | 0.1 | 0.1 |
| selenoamino acid metabolism | NA | NA | NA | NA | NA | NA | 1.3 | 0.1 | 0.2 | 1.6 | 0.1 | 0.3 |
| spermidine and spermine biosynthesis | 1.4 | 0.1 | 0.0 | NA | NA | NA | 1.6 | 0.1 | 0.1 | NA | NA | NA |
| starch and sucrose metabolism | NA | NA | NA | NA | NA | NA | 1.4 | 0.1 | 0.5 | NA | NA | NA |
| steroid hormone biosynthesis | 2.5 | 0.2 | 0.4 | 2.5 | 0.1 | 0.3 | NA | NA | NA | 2.3 | 0.2 | 0.7 |
| steroidogenesis | 1.7 | 0.2 | 0.4 | NA | NA | NA | NA | NA | NA | NA | NA | NA |
| sulfate/sulfite metabolism | NA | NA | NA | NA | NA | NA | 1.6 | 0.1 | 0.4 | NA | NA | NA |
| sulfur metabolism | 2.2 | 0.1 | 0.0 | NA | NA | NA | 3.4 | 0.1 | 0.1 | 1.6 | 0.1 | 0.1 |
| taurine and hypotaurine metabolism | NA | NA | NA | NA | NA | NA | 1.6 | 0.1 | 0.0 | NA | NA | NA |
| toluene degradation | 1.5 | 0.2 | 0.4 | 1.3 | 0.1 | 0.3 | 2.0 | 0.1 | 0.2 | NA | NA | NA |
| tropane, piperidine and pyridine alkaloid biosynthesis | 1.4 | 0.2 | 0.4 | 1.1 | 0.1 | 0.5 | 1.9 | 0.1 | 0.4 | NA | NA | NA |

| | | | | | | | | | | | | |
|---|---|---|---|---|---|---|---|---|---|---|---|---|
| tryptophan metabolism | 2.7 | 0.1 | 0.0 | 2.0 | 0.1 | 0.0 | 4.6 | 0.0 | 0.0 | 1.7 | 0.1 | 0.0 |
| tyrosine metabolism | 3.1 | 0.2 | 0.1 | 3.8 | 0.1 | 0.1 | 5.4 | 0.1 | 0.1 | 4.1 | 0.1 | 0.1 |
| ubiquinone and other terpenoid-quinone biosynthesis | NA | NA | NA | 1.1 | 0.2 | 0.6 | NA | NA | NA | 1.3 | 0.1 | 0.4 |
| ubiquinone biosynthesis | NA | NA | NA | NA | NA | NA | 1.1 | 0.1 | 0.4 | NA | NA | NA |
| urea cycle | NA | NA | NA | NA | NA | NA | 1.6 | 0.1 | 0.0 | NA | NA | NA |
| urea cycle/amino group metabolism | 3.5 | 0.1 | 0.0 | 1.7 | 0.1 | 0.0 | 3.8 | 0.0 | 0.0 | 1.1 | 0.1 | 0.1 |
| valine, leucine and isoleucine biosynthesis | 1.2 | 0.2 | 0.1 | 1.1 | 0.1 | 0.2 | 1.5 | 0.1 | 0.2 | NA | NA | NA |
| valine, leucine, and isoleucine degradation | 1.9 | 0.2 | 0.1 | 1.3 | 0.1 | 0.1 | NA | NA | NA | NA | NA | NA |
| vitamin b3 (nicotinate and nicotinamide) metabolism | 1.5 | 0.2 | 0.1 | NA | NA | NA | 1.4 | 0.1 | 0.0 | NA | NA | NA |
| vitamin b9 (folate) metabolism | NA | NA | NA | 1.6 | 0.1 | 0.2 | NA | NA | NA | NA | NA | NA |
| warburg effect | NA | NA | NA | NA | NA | NA | NA | NA | NA | 1.5 | 0.1 | 0.1 |
| xenobiotics metabolism | 3.3 | 0.2 | 0.1 | 1.0 | 0.1 | 0.1 | 1.9 | 0.1 | 0.0 | NA | NA | NA |
| xylene degradation | 2.0 | 0.2 | 0.2 | 1.7 | 0.1 | 0.4 | 2.7 | 0.1 | 0.1 | 2.2 | 0.1 | 0.0 |
| zeatin biosynthesis | NA | NA | NA | 1.2 | 0.1 | 0.0 | NA | NA | NA | NA | NA | NA |

**Supplemental Table 4.** Number of statistically significant associations between prenatal $NO_x$ exposure and metabolite intensity at various significance thresholds, including only untargeted metabolites which were identified in both cohorts.

| | Atlanta African American Maternal-Child Cohort | | | | | | Mother's Milk Study | | | | | |
|---|---|---|---|---|---|---|---|---|---|---|---|---|
| **Exposure** | **P<0.05** | **P<0.01** | **P<0.005** | **P<0.0005** | **$P_{BH}$<0.20** | **$P_{BH}$<0.05** | **P<0.05** | **P<0.01** | **P<0.005** | **P<0.0005** | **$P_{BH}$<0.20** | **$P_{BH}$<0.05** |
| **Cumulative Pregnancy $NO_x$** | 133 | 35 | 17 | 5 | 5 | 0 | 104 | 25 | 16 | 7 | 12 | 4 |
| **1st Trimester $NO_x$** | 138 | 30 | 18 | 1 | 1 | 0 | 91 | 21 | 12 | 1 | 0 | 0 |
| **2nd Trimester $NO_x$** | 115 | 31 | 16 | 3 | 2 | 0 | 110 | 28 | 17 | 5 | 6 | 2 |
| **3rd Trimester $NO_x$** | 105 | 24 | 16 | 1 | 0 | 0 | 87 | 23 | 14 | 6 | 7 | 5 |

**Supplemental Table 4.** Results were generated using linear models, where the outcome was logged metabolite intensity. Models for the Mother's Milk Study were adjusted for infant and sex, maternal education, season of study visit, maternal age, and maternal pre-pregnancy body mass index. Models for the Atlanta African American Maternal-Child cohort were additionally adjusted for alcohol, tobacco, and marijuana use during pregnancy. Only untargeted metabolites which could be measured in both cohorts were included.
